# Cell differentiation, aging, and death in spatially organized yeast communities: mechanisms and consequences

**DOI:** 10.1038/s41418-025-01485-9

**Published:** 2025-03-29

**Authors:** Zdena Palková, Libuše Váchová

**Affiliations:** 1 Department of Genetics and Microbiology, Faculty of Science, BIOCEV, Charles University, Prague, Czech Republic; 2 Institute of Microbiology of the Czech Academy of Sciences, BIOCEV, Prague, Czech Republic

**Keywords:** Microbiology, Development, Ageing

## Abstract

Cell death is a natural part of the development of multicellular organisms and is central to their physiological and pathological states. However, the existence of regulated cell death in unicellular microorganisms, including eukaryotic and prokaryotic microbes, has been a topic of debate. One reason for the continued debate is the lack of obvious benefit from cell death in the context of a single cell. However, unicellularity is relative, as most of these microbes dwell in communities of varying complexities, often with complicated spatial organization. In these spatially organized microbial communities, such as yeast and bacterial colonies and biofilms growing on solid surfaces, cells differentiate into specialized types, and the whole community often behaves like a simple multicellular organism. As these communities develop and age, cell death appears to offer benefits to the community as a whole. This review explores the potential roles of cell death in spatially organized communities of yeasts and draws analogies to similar communities of bacteria. The natural dying processes in microbial cell communities are only partially understood and may result from suicidal death genes, (self-)sabotage (without death effectors), or from non-autonomous mechanisms driven by interactions with other differentiated cells. We focus on processes occurring during the stratification of yeast colonies, the formation of the extracellular matrix in biofilms, and discuss potential roles of cell death in shaping the organization, differentiation, and overall physiology of these microbial structures.

## Facts

- In nature, microbes typically form spatially organized multicellular communities, such as colonies and biofilms.
- These microbial communities are composed of differentiated cell subpopulations, each performing specialized functions.
- In yeast colonies, cell death occurs within specific subpopulations, benefiting the community by supplying nutrients and other essential compounds.
- The mitochondria-regulated retrograde pathway (RTG) plays a key role in repressing cell death in specific subpopulations within yeast colonies.
- The role of cell death in shaping the three-dimensional structure of yeast communities remains largely unexplored.

## Open questions

- Which intracellular and extracellular signals drive cell death in spatially organized yeast communities?
- Where, why, and how do cells die within yeast biofilms?
- What factors and molecular mechanisms suppress the RTG signaling pathway and trigger cell death in differentiated yeast colonies?
- What techniques can accurately identify and characterize dying yeast cells within colonies and biofilms without disrupting their structure?

## Introduction

Programmed cell death (PCD) and regulated cell death (RCD) are essential processes in metazoan development, where they play critical roles in eliminating cells during organ formation and removing damaged or pathogenic cancer cells [1]. In metazoans, distinct types of cell death have been characterized both morphologically and mechanistically [2]. As new conditions and mechanisms that cause cell death are discovered across different organisms, the classification of these death processes has become increasingly complex [3]. Here we apply the term cell death to discuss the loss of cells in naturally occurring, spatially structured microbial communities and in laboratory conditions intended to mimic such scenarios, where evidence suggests that cell death is an active, physiologically relevant process.

Applying the terms PCD/RCD in unicellular microbes, particularly in yeast, raises different questions. Yeasts are eukaryotes, yet studies of yeast proteins homologous to mammalian factors involved in PCD/RCD often reveal divergent functions. A typical example is the *S. cerevisiae* metacaspase Mca1p (also known as Yca1p, yeast caspase), which has different substrate specificities than mammalian caspases [4]. Rather than playing a role in yeast cell death, Mca1p seems to be involved in proteostasis, particularly in disaggregating protein aggregates [5, 6]. Currently, knowledge about molecular machineries involved in cell death process in yeast is limited. A recent study, however, identified a cell death effector pathway in yeast involving the AP-3 vesicle trafficking complex that transports the protein kinase Yck3 to the vacuolar membrane and leads to vacuole/lysosome membrane permeabilization followed by cell death [7]. This finding supports earlier studies highlighting the importance of vacuolar components in yeast cell death processes [8, 9]. There is also ongoing debate regarding whether and how yeast cell death under particular conditions (such as nutrient deprivation, exposure to different external compounds, or specific developmental processes) is regulated by intrinsic or extrinsic factors. For example, numerous studies have shown the effect of acetic acid on yeast cell death, but the detailed mechanisms of cell death remain unclear [10]. Much of the recent research on microbial cell death has focused on bacterial cell suicide triggered by phage infection, an example of cell death as an evolutionary adaptation [11]. However, the more challenging studies of physiological (vs. pathological) microbial cell death, which may be much more prevalent among life-forms, remain understudied.

Here, we address a fundamental, frequently discussed question: How could cell death be beneficial for a unicellular organism like yeast? In yeast, as in bacteria, cell death is synonymous with organismal death [12, 13], seemingly offering no advantage to the unicellular organism that dies. However, yeast and other microbes rarely exist as single cells in nature. Instead, they typically form multicellular communities that are often precisely organized, with various social interactions occurring among microbial cells [14–21]. Within these communities, microbial cells often diversify into specialized cell subpopulations with distinct morphologies, metabolic profiles, and regulatory pathways. These cells are spatially positioned within the community and perform specialized functions (e.g., [16, 17, 20–23]). Thus, microbial cells can be considered components of spatially organized multicellular communities, where the death of a fraction of cells could benefit the overall population for multiple reasons.

**Fig. 1:**
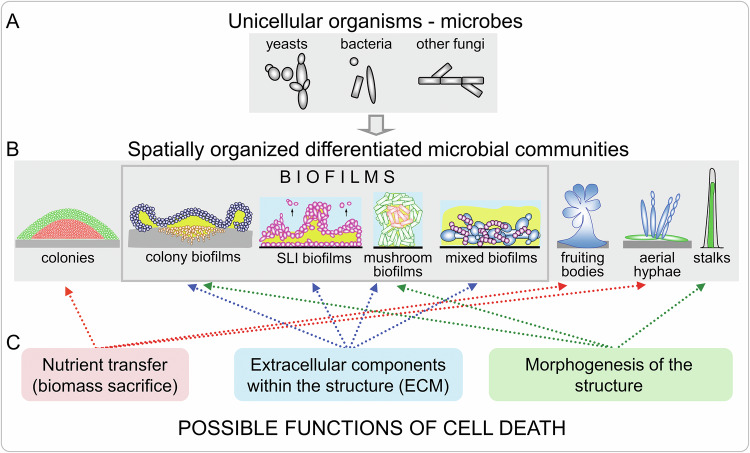
Examples of spatially structured microbial communities and the potential role of cell death in their development, differentiation and the emergence of specific properties. **A** Many microorganisms including yeasts, bacteria, and other fungi naturally form spatially structured communities composed of variably shaped cells, including spherical yeast cells, which also form hyphae and pseudohyphae, bacterial coccoid, rod-like, and mycelial cells, and typical fungal mycelial forms. **B** Models of diverse spatially structured communities (vertical slices, side views). From the left, colonies of the yeast *S. cerevisiae* on agar consist of upper (U) cells (green) and lower (L) cells (red). Colony biofilms of wild *S. cerevisiae* on agar with aerial yeast-shaped cells (dark blue), pseudohyphae invading agar (ochre) and extracellular matrix (ECM, yellow) that contributes to biofilm formation; solid-liquid interface (SLI) biofilms of wild *S. cerevisiae* (violet) with ECM (yellow) growing on plastic (black line); mushroom-shaped SLI biofilms of Gram-negative bacteria with living (green) cells surrounding a core of dying (red) cells; mixed biofilms of yeast *C. albicans* (blue) and bacteria *S. aureus* (purple) with ECM (yellow). Fruiting bodies of *Myxobacteria* (blue); aerial hyphae of *Streptomyces* (blue) arising from vegetative mycelium (green); and *S. cerevisiae* stalks with living cells and spores inside (green) and dying cells forming an envelope (black line). **C** Possible functions of cell death in the various types of spatial communities. Arrows indicate spatial structures in which a specific role of cell death is expected based on their properties discussed in the text.

In recent decades, significant progress has been made in understanding microbial multicellularity, with research focusing on diverse spatial structures formed by microbial populations, including different types of biofilms, colonies, fruiting bodies, mats, and other less common structures (Fig. 1) [16, 18, 20, 24]. The spatial arrangement enables the creation of gradients of nutrients, waste products, and signaling molecules that contribute to site-specific cell differentiation into functionally specialized cell subpopulations with distinct metabolism and regulation [16, 18, 20–23]. Despite increasing knowledge about the internal organization of yeast spatial structures, understanding of molecular mechanisms of cell death within these structures remains insufficient, making it difficult to classify the types of cell death that occur. Nevertheless, in some cases, we can determine whether yeast cell death is genetically modulated and regulated (either delayed or accelerated). In contrast, bacterial spatial structures have been more extensively studied, and specific mechanisms of cell-cell interactions and factors directly involved in bacterial cell death (often referred to as regulated autolysis) have been identified. In addition, relatively well-characterized mechanisms of bacterial innate immunity (Abi systems) cause abortive infection to prevent the spread of phage infections within bacterial populations. These systems serve as a defense mechanism, sacrificing infected cells to protect the larger community [25]. Whether similar mechanisms play a significant natural role in biofilm dynamics remains to be determined.

In this review, we focus on the roles of cell death in the development of spatially structured yeast populations, in the context of other features of the community, such as the presence of specific cell types, their metabolism, regulation, and function. Since bacterial research in this field is more advanced, we also compare aspects of these processes with insights gained from similar structures formed by bacteria. We provide selected examples of bacterial cell death that is specifically regulated or is part of a bacterial life cycle program, such as in the fruiting bodies of *Myxobacteria*. We believe that insights from studies on bacterial cell death will help advance research on cell death in spatial structures of eukaryotic yeasts and will lead to the identification of new mechanisms.

## Roles of cell death in spatially organized communities

Current knowledge indicates that cell death may serve different roles in spatially organized yeast and bacterial populations, such as biofilms, colonies, stalks, and fruiting bodies (Fig. 1). The potential roles of cell death, discussed in the following sections, include: i) adaptive cell death associated with biomass sacrifice for intercellular nutrition cross-feeding, where nutritive compounds from dying cells are used by other cells in the spatial population; ii) contribution of cell death to extracellular matrix (ECM) composition and iii) role of cell death in morphogenesis of spatial structures.

These potential roles of cell death vary in importance across different types of spatial populations due to differences in population structure, complexity, composition, organization, development, and differentiation. The arrangement and development of spatial microbial structures exhibit common features, such as the presence of spatially positioned differentiated cells and reproducible development under specific growth conditions and strains. However, different structures also show diversity in their characteristics, such as extracellular matrix (ECM) production typical for biofilms, in responses to varying conditions like nutrient availability, and differences among yeast or bacterial genera or strains.

In yeast, most knowledge about cell differentiation, metabolism, and regulation of specialized cells comes from studies of smooth colonies and wrinkled biofilms of *Saccharomyces cerevisiae* and *Candida albicans*. These structures form at the semisolid agar-air interface (smooth colonies and wrinkled colony biofilms) or at the solid/semi-solid-liquid interface (wrinkled SLI biofilms); differences in their properties are described below.

## The diversity of yeast spatial structures: colonies and biofilms

Two types of yeast biofilms that are most studied, wrinkled colony biofilms and wrinkled SLI biofilms, differ primarily in their growth conditions. Colony biofilms form on agar plates, while SLI biofilms typically develop at the bottom of plastic chambers, such as microtiter plates, in liquid media. Strains that form colony biofilms often form also SLI biofilms, demonstrating their ability to adhere to solid/semi-solid surfaces and produce ECM, as documented for the *S. cerevisiae* wild strain BR-F [26–28].

Three types of smooth colonies are distinguished by their origin and strain characteristics: transient smooth colonies of biofilm-forming strains in nutrient-rich environments, smooth colonies of domesticated strains that form under all nutrient conditions, and smooth colonies of laboratory strains that have lost the ability to switch to a biofilm lifestyle.

The organization of yeast spatial structures is primarily determined by genetic and epigenetic programs active in specific yeast strains under given conditions. For example, some strains of *S. cerevisiae* form biofilms, while others form smooth colonies under the same growth conditions [29, 30]. Additionally, “lifestyle” of cells leading to biofilm formation can be influenced by environmental factors such as nutrient availability or stress exposure. Some *S. cerevisiae* strains isolated from nature, such as the BR-F strain, form wrinkled colony biofilms on agar and wrinkled SLI biofilms on plastic [18, 27, 28, 30]. However, in the presence of rich nutrient sources (e.g., glucose), these strains transiently repress biofilm characteristics, such as ECM production, and form smooth colonies instead [29]. As the colony ages and nutrients are depleted, the cells revert to their wild phenotype, resuming biofilm characteristics and altering the structure morphology back to a biofilm form [29].

A phenotypically similar but heritable change from biofilm formation to colony formation is a phenotypic switching known as domestication [17, 30–34]. Domesticated yeast cells derived from the wild *S. cerevisiae* strain BR-F have switched off their biofilm properties and form smooth colonies regardless of nutrient conditions, i.e., even in the absence of glucose. Domesticated cells appear in colony biofilms with a frequency reaching ~7% on glucose and ~2% on respiratory media within approximately 20 days [35]. Although the mechanisms underlying this domestication process are not fully understood, several have been proposed, including DNA rearrangements, the involvement of prions, and epigenetic reprogramming [35–37]. For instance, differences in the histone deacetylase Hda1 have been observed between BR-F strain and domesticated strain BR-S [35]. However, the mechanisms appear to vary between strains. For example, DNA rearrangements leading to aneuploidy were involved in the domestication of the *S. cerevisiae* sake strain UC5, but not in the BR-F strain [35, 37].

The domestication of the BR-F strain is reversible, so that a return to the biofilm properties, the so-called “feralization”, can occur. Feralization (recognizable by the appearance of cells that form wrinkled biofilms again) of the domesticated BR-S strain occurs at a low frequency (1-5%) under nutrient-limited conditions after approximately 30-80 days, with the frequency increasing to 15-25% after 100 days [35]. In contrast, feralization has not been observed in laboratory *S. cerevisiae* strains, which exhibit a stable domesticated state and have lost the ability to “go wild,” likely due to accumulated mutations [38].

## Cell death and nutrient flow in yeast colonies: mechanisms and implications

### Cell death and nutrient distribution in smooth colonies of *S. cerevisiae* laboratory strains

Smooth colonies of *S. cerevisiae* laboratory strains consist of densely packed cells with minimal intercellular spaces, as observed through environmental scanning electron microscopy (ESEM) and two-photon excitation confocal microscopy (2PE-CM) of vertical colony cross-sections [30, 39]. Cell growth in these colonies is vertically oriented: the oldest cells are located in the lower regions, while the younger cells are found at the margins and in the upper parts [40]. This was documented by the distribution of GFP-labeled cells in chimeric giant colonies (formed from a suspension of GFP-labeled and GFP-unlabeled cells) and by tracking cells stained with AlexaFluor488 5-TFP used for giant colony inoculation [40].

Colonies growing on a complete respiratory medium undergo several developmental phases, each associated with extensive metabolic reprogramming, detectable changes in extracellular pH (using a pH indicator in the plate), and cell differentiation in different parts of the colony [41–45]. Developmental phases are similar in microcolonies (arising from a single cell) and giant colonies (arising from a cell suspension), although the timing of these phases varies depending on the number and size of colonies on the plate [41, 44].

**Fig. 2:**
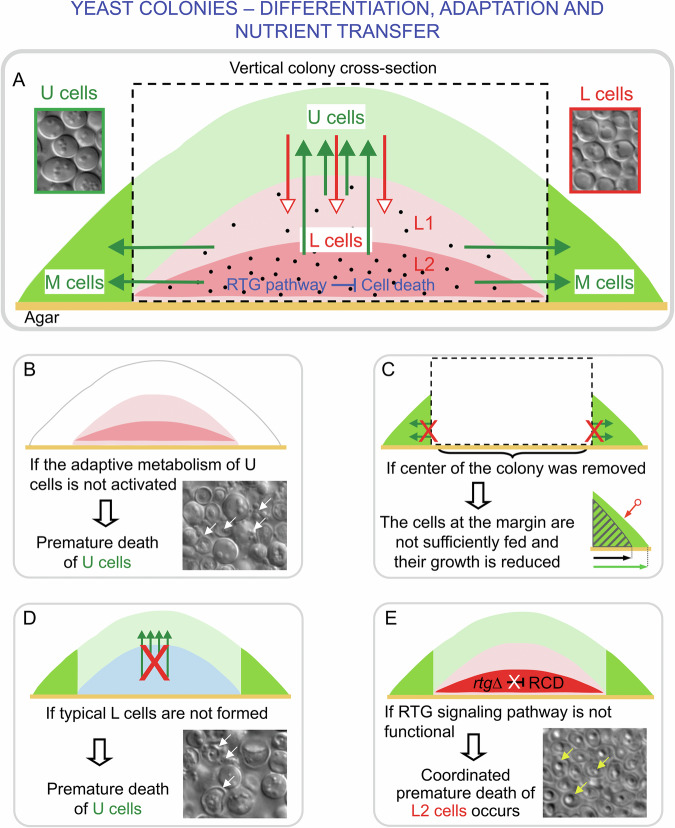
Biomass sacrifice and cross-feeding between differentiated subpopulations in yeast colonies. **A** Schematic of a differentiated *S. cerevisiae* colony. Arrows indicate the direction of nutrient flow from lower (L) cells (L→U/M, solid green arrows) and the flow of waste products from upper (U) cells for recycling in L cells (open red arrows). Microscopy images of U and L cells are shown (DIC visualization). The central area of a yeast colony that is slated for experimental removal in (**C**) (dashed box) consists of U cells (light green) and two subpopulations of L cells, L1 cells (light pink) and L2 (dark pink); outside this region are the marginal (M) cells (intense green). Cell death depicted by the density of black dots is higher in L2 than in L1 cells in healthy colonies. The RTG signaling pathway in L2 cells represses premature cell death. **B**–**E**. Documented interactions between yeast cell types in differentiated *S. cerevisiae* colonies. **B** Premature U cell death occurs if their adaptive metabolism is not activated [41], such as autophagy-deficient U cells, resulting in defective development and some cell death (white arrows in microscopy (DIC) image mark intracellular material released from dying or lysed cells with visible cell walls). **C** Removal of all cells from the central area of a 16-day-old giant colony (dashed box, as in A), results in reduced nutrient availability from L cells (red X’s), and the total marginal cell area (green) in unexcised control colonies (red-circle and light green arrow) is reduced by 25% in excised colonies after 6 days (hatched area, black arrow) [47]. **D** Furthermore, the absence of normal L cells leads to premature death of U cells, probably due to insufficient nutrients (white arrows mark dead/dying U cells in a *cat8* gene-deficient colony lacking normal L cells for support) [41]. **E** Premature and coordinated L2 cell death is enhanced in colonies lacking a functional RTG signaling pathway (*rtg*Δ mutants) [43]. Microscopy (DIC) image of dead/dying L2 cells (yellow arrows) with the characteristic shrunken phenotype as described in [47] in *rtg*Δ colonies [43].

Both vertical and horizontal differentiation occurs within these colonies, resulting in the formation of distinct cell subpopulations (Fig. 2) [20, 41, 44–47]. These specialized cell types are spatially localized and perform unique functions that enable cooperation and coordination within the colony. This organization promotes the overall development of the colony, supports the long-term viability of cells in advantageous regions, and optimizes the colony potential to expand and access new nutrient sources in natural environments. The latter is also documented by the ability of cells with novel properties to expand specifically within the U cell layer [48].

#### Distinctive traits of differentiated cells in colonies: morphology, metabolism, and viability of U, M and L cells

Volatile ammonia is a crucial signal that regulates the acidic-to-alkali developmental phase transition in colonies, occurring around 8-10 days in giant colonies (6 per 9 cm plate) and around 4 days in microcolonies (5 × 10^3^ per 9 cm plate) [42, 44]. The transition is associated with the vertical differentiation of central parts of colonies into distinct layers containing two main cell types (Fig. 2A): U cells, located in the upper colony layers, and L cells, residing in the middle and lower layers [41, 44]. As colonies develop, the border between U and L cells sharpens, making the two subpopulations distinct in fully differentiated colonies (Fig. 2A) [41, 43, 49]. Ammonia plays a role in this differentiation process, as it can induce the formation of U cells in the upper layers of young, not-yet differentiated colonies [41].

A combination of microscopy, OMICs, enzyme activity measurements, and analyses of specific regulators (TORC1, Gcn4p, and RTG) has shown that U and L cells differ in morphology, gene expression, metabolism, activity of different regulators, and viability [41, 43, 44, 49, 50]. Long-lived U cells exhibit unique adaptive metabolism, with features of metabolically active cells, including active translation, amino acid metabolism, glycolysis, and an active TORC1 pathway (evidenced by Gat1p cytosolic localization) [41, 44, 51]. They also display characteristics of cells entering the stationary phase, such as a thick cell wall resistant to stressors, accumulation of glycogen and trehalose, active autophagy, and an active Gcn4p transcription factor [41, 49, 52]. In colonies of mutant strains where some of these processes, such as autophagy, are inactive, earlier death of U cells is observed, while L cells remain unaffected (Fig. 2B) [41].

Conversely, L cells are stress-sensitive, have higher ROS levels than U cells, a zymolyase-sensitive cell wall, do not accumulate glycogen and trehalose, and have inactive TORC1 and Gcn4p pathways [41, 44, 49, 52]. They also lack autophagy and behave like starving, stressed cells, expressing numerous hydrolytic enzymes (including those that hydrolyze the cell wall) and proteasomal proteins [41]. Over time in aging colonies, L cells show decreased levels of intracellular free amino acids compared to U cells, with different amino acid compositions between the two. U cells contain higher levels of glutamine, glutamate, and arginine, and lower levels of alanine, GABA, and lysine than L cells [41]. Notably, only U cells produce ammonia [41].

A key difference between U and L cells is their respiratory capacity [41, 44]. U cells reduce respiration, possess fewer swollen mitochondria with reduced cristae, and decrease the expression of mitochondrial OXPHOS and ATP synthase proteins. L cells, however, can respire when separated from colonies, have cristated mitochondria, and express higher levels of OXPHOS and ATP synthase proteins.

A third cell type, M cells, is located at the colony margin (Fig. 2A). Transcriptomic comparisons revealed that M cells share several expression features with U cells but also have unique expression properties, such as those related to cell cycle genes [53]. Growing (dividing) cells were found at the colony margin and slowly-growing cells within the U cell layer, which thickened from 9 to 28 days by ~35%. The presence of dividing cells in aged colonies was demonstrated using a thermosensitive *cdc3-1* mutant. When dividing cells of this mutant are exposed to a non-permissive temperature, they reach a specific terminal phenotype [54], which was visible on colony cross-sections. In contrast, no such dividing *cdc3-1* cells were observed within the L cell layer [41].

#### Interaction between differentiated cell types: biomass sacrifice and nutrient transfer

Early studies revealed that dying cells are present in the center of the colony and that the presence of cells in the center supports the growth of cells at the margin in old colonies. First, comparisons between central cells and margin M cells (Fig. 2A) in giant colonies aged 5-28 days showed a significant increase in the number of cells with cell death features in the colony center, whereas such cells were almost absent among M cells [47]. These analyses identified dying cells based on high ROS levels, DNA breaks, phosphatidylserine relocalization to the cell surface, and the presence of dead cells with a shrunken phenotype, characterized by a visible cell wall and reduced cellular content [47]. In colonies lacking ammonia signaling (e.g., in *sok2* mutants), cells with these death-related features were also found in greater numbers at the colony margin compared to wild-type colonies. However, the distribution of dying cells between the colony center and margin was independent of the putative yeast cell death proteins Mca1 and Aif1 [47, 55, 56]. Second, removal of the entire central portion of differentiated 16-day-old giant colonies (containing U and L cell subpopulations and already dead cells in the center) caused a marked reduction in the growth of M cells at the margin (Fig. 2C), despite intact agar nutrient sources being available as in control colonies [47]. These findings demonstrated that central cells are essential for sustaining growth at the colony margin in older, differentiated colonies. It was hypothesized that nutrients and other compounds released from dead central cells provide essential resources for the younger M cells at the margin [47].

Later studies of vertical stratification of the colony center into U and L cells revealed that ~50% of all U cells remained viable in 50-day-old colonies, compared with only ~10% of L cells being viable, indicating that L cells contribute to the increased number of dying central cells as the colony ages [41]. Based on the metabolic differences between U and L cells, a model of nutrient and waste product cycling between these two cell types was proposed [41], which resembles the Cori and glutamine-ammonia cycles found between tumor cells and cells in normal tissues (liver and muscle cells) of the tumor-affected organism [41, 57]. According to this model, L cells recycle waste products and provide nutrients to U cells for their slow growth [41]. Supporting this model, higher carbohydrate release was detected in L cells compared to U cells, which have a greater capacity to uptake glutamine and glucose and release ammonia [41, 51]. U cell viability appears dependent on glycolysis, as indicated by the high number of dead cells and reduced growth of the U cell layer in giant colonies treated with glycolytic inhibitors (2-deoxy-D-glucose and iodoacetamide) and the death of U cells in *pfk2* mutant colonies [41, 51]. Since colonies grow from beginning on respiratory medium without glucose, L cells likely serve as a carbohydrate source for U cell glycolysis. In mutant strains lacking typical L cells (e.g., *coa1*, *ndi1*, and *cat8* mutants), dead cells appear early in the U cell layer (in 15-day-old giant colonies) (Fig. 2D), likely due to the lack of nutrients normally supplied by L cells [41]. These nutrients could come from two sources: those gradually released from still-lived L cells and those from dying L cells. This scenario suggests that U cells are gradually fed at the expense of L cells during their long-term coexistence within the colony.

Two prerequisites are crucial for U cell - L cell interaction and cross-feeding scenario. First, the metabolic properties and nutrient transport mechanisms in aging L cells should prevent these cells from reutilizing the nutrients they release. Consistent with this requirement, L cells exhibit lower glutamine and glucose uptake but higher carbohydrate and glucose release compared to U cells [41, 51]. Additionally, the metabolic profile of L cells is adapted to facilitate nutrient transfer to U cells. This adaptation includes the upregulation of fructose-1,6-bisphosphatase, a key gluconeogenesis enzyme, and enzymes from specific segments of the TCA and glyoxylate cycles, which synthesize 2-oxoglutarate and glutamate-precursors of glutamine [51]. These findings are supported by transcriptional profiles and enzyme activity analyses [41, 51]. Second, maintaining a proper balance between dying L cells, which are presumed to provide higher amounts of nutrients short-term, and the remaining living L cells, which could supply low-levels of nutrients long-term, should be critical to colony longevity. An imbalance in this ratio could disrupt colony structure and lifespan. For instance, excessive or premature L cell death could lead to a rapid depletion of L cell resources, leaving U cells inadequately supported. Conversely, insufficient L cell death could result in insufficient nutrient release, impairing U-cell survival. Unlike U cells, L cells do not divide in differentiated colonies, as evidenced by experiments with the *cdc*3-1 mutant [41]. This suggests that the L cell subpopulation is not regenerable, further underscoring the importance of balanced L cell survival and death for colony viability.

#### Regulation of L cell viability by the retrograde signaling pathway

The mitochondrial retrograde (RTG) signaling pathway transmits information about reduced or altered mitochondrial functions to the nucleus, inducing specific genes and reprogramming cellular metabolism [58–60]. This pathway is well-known for reprogramming cells with dysfunctional mitochondria and activating anaplerotic metabolism in yeast liquid cultures. This reprogramming enables cells to synthesize metabolic building blocks like glutamate and glutamine via the glyoxylate cycle and peroxisomal fatty acid beta-oxidation [58–60]. However, in colonies, the RTG pathway is more complex than in liquid cultures and functions differently in subpopulations of U versus L cells to regulate different targets [43].

Rtg activators are expressed and active in both U and L cells of wt colonies [43]. When the RTG pathway is disrupted (e.g., by deleting genes encoding the RTG pathway activators Rtg1p-Rtg3p), a massive death of a specific sub-fraction of L cells, called L2 cells (localized to the lower part of L cells), occurs (Fig. 2E) [43]. These L2 cells exhibit a coordinated cell death, reaching a shrunken dead phenotype, so that almost all these cells are dead in 17-day-old giant colonies. No cell death is observed in *rtg*Δ U cells or in the upper layer sub-fraction of L cells, called L1 cells [43]. Proteomics combined with microscopy of GFP-tagged proteins in colonies revealed other functions of the RTG pathway specific to U or L1 cells [43, 50]. In U cells, the RTG pathway positively regulates genes involved in ammonia transport (*ATO*), in the synthesis and transport of acetyl-CoA, and the metabolism of amino acids, and it negatively regulates mitochondrial translation, possibly contributing to the repression of mitochondrial respiration [50]. In L1 cells, the RTG pathway induces the expression of a few genes, including *CIT2*, a marker of RTG pathway activation in the context of anaplerotic metabolism in liquid cultures [43, 50]. These differences in RTG signaling contribute to the functional diversification of U and L1 subpopulations, which is critical for colony development and the maintenance of U cell longevity.

Thus, the RTG pathway activity in L2 cells has a role in preventing premature death of these cells, potentially controlling the ratio of dying cells to living cells that can process waste products of U cell metabolism and provide nutrients to U cells. Although yet unproven, the premature death of L2 cells in RTG pathway-defective colonies is thought to significantly disrupt the cycling of waste and nutrients between U and L cells, thereby compromising the long-term survival of the U cells.

#### Altruism or predation?

The question of whether yeast cell death represents an altruistic and adaptive evolutionary process (altruistic cell sacrifice) has been widely debated in the context of microbial interactions [11, 12]. This question also applies to differentiated colonies. Is the cycling of nutrients and waste between U and L cells—where L cells appear to support U cells at their own expense—an altruistic sacrifice by L cells, or could it represent a form of predatory behavior by U cells, forcing L cells to release nutrients in a manner similar to fratricide described in bacterial biofilms? At present, there is no data to determine whether L cell death utilizes a dedicated molecular cell death machinery (none has been identified to date) or whether it involves a sabotage-like mechanism, where key pro-survival metabolic processes are blocked [61]. Another unresolved question is whether L cell death occurs autonomously (without external signals or influence from other cell types in the colony) or whether it is induced non-autonomously by signals from other cells, such as U cells. In the latter case, L cell death would be initiated by intercellular signaling, similar to the non-autonomously induced cell death described in metazoans like *Drosophila*, *Caenorhabditis elegans*, and mammals. In these systems, intercellular signaling can trigger cell death, including apoptosis [62].

Currently, there is no definitive evidence supporting either autonomous L cell differentiation and death or non-autonomous induction mediated by other cells in the colony. However, existing knowledge suggests that interactions between U and L cells - and possibly between their precursors - play a crucial role in these processes. To better understand cell interaction and differentiation, several complex and interrelated factors must be taken into account, as described below.

i. *Nutrient accessibility*: The fate of cells in the colony does not simply depend on the accessibility of nutrients from the nutritive agar. Long-lived U cells are located in the upper parts of colonies, far from the agar, a primary nutrient source. In contrast, starving and gradually dying L cells are found in the lower and middle parts, closer to the nutrients in the agar (Fig. 2A). Similarly, U cells with good access to air decreased mitochondrial respiratory capacity, while L cells with less access to air are capable of respiration. Decreased respiration likely helps to reduce ROS levels in U cells under nutrient-limiting conditions.
ii. *Vertical cell growth*: In developing colonies, older cells are at the bottom (near the nutrients), and younger cells appear progressively at higher positions in the growing colony [40], far from the nutrients. This means that cells that differentiate into L cells are chronologically older than those differentiating into U cells. The chronological age of L cells may contribute to their gradual death despite their proximity to nutrients, but this hypothesis is difficult to test conclusively.
iii. *Ammonia signaling*: Among extracellular metabolites detected in colonies, ammonia has shown a clear signaling role. Gaseous ammonia acts as a long-distance quorum-sensing molecule, inducing its own production in cells in young colonies and synchronizing the development of nearby colonies that are within the ammonia diffusion range [63]. In these young colonies, ammonia induces the expression of genes typically expressed in U cells, such as ATO genes, and contributes to U cell formation [41, 64]. However, in fully differentiated colonies containing distinct U and L cell subpopulations, ammonia production is restricted to U cells [41]. This observation suggests that cells in lower regions (which later differentiate to L cells) are not induced to produce ammonia during differentiation. This distinct production profile points to differences in how cells of varying chronological ages respond to ammonia signaling, leading to their reprogramming into distinct cell fates. Interestingly, studies on yeast grown in liquid cultures have shown toxic effects of ammonium on aging yeast cells, raising the possibility that ammonium may also be a death stimulus in differentiated colonies [65, 66].

Further studies are needed to clarify whether the differentiation and death of L cells are autonomously initiated or induced by U cells, and to better understand the role of ammonia in this process.

### Cell differentiation and interaction in sporulating *S. cerevisiae* colonies

*S. cerevisiae* laboratory strains grown on acetate medium form smooth colonies. In these colonies formed by diploid strains, some cells in the upper regions undergo meiotic division to form spores (sporulating cells, ~50%), while cells in the lower regions do not sporulate and are thought to contribute to developing spores by acting as feeder cells [67–70]. The differentiation process into sporulating and feeder cell subpopulations is gradual until sharply delineated sporulating and feeder cells are formed after ~4 days [67–70]. The positions within colonies of these sporulating and feeder cells roughly correspond to position of U cells and L cells, respectively. However, it is important to note that, despite their similar positions, the properties of sporulating cells compared to U cells, and feeder cells compared to L cells, can differ significantly due to the distinct nutrient conditions under which they develop.

Regulatory circuits involving autonomous (within each cell subpopulation) and non-autonomous (between subpopulations) signals control the formation of sporulating and feeder cells. These circuits include the cell wall integrity (CWI) pathway, involving the Mpk1/Slt2 protein kinase-Rlm1p regulatory loop in lower region cells (feeder cells), and the Ime1-alkaline signaling Rim101p regulatory loop in upper region cells (sporulating cells) [68–70]. These pathways are essential for the development of each cell type and are coordinated by not-yet-identified non-autonomous signals [68]. This coordination is evidenced by mutant analyses showing that disruption of regulation in one cell type affects the development of the other. For example, colonies with *RLM1* deletion did not form a typical feeder cell layer, and sporulation was less efficient, lacking a clear sporulation pattern [69]. This evidence also suggests that feeder cells are important for the formation of sporulating cells, presumably in part by providing nutrients to sporulating cells [69]. Further studies are needed to test this hypothesis.

### Cell death and nutrient transfer in *Myxobacteria* fruiting bodies and *Streptomyces* aerial hyphae

Among the earliest examples of cell death in microbes associated with biomass sacrifice and cross-feeding is the development of fruiting bodies and sporulation in *Myxobacteria*, best studied in *Myxococcus xanthus* [71, 72]. *Myxobacteria* are predatory bacteria that form organized fruiting bodies containing about 10^5^ cells, where cell differentiation and precise spatiotemporal arrangement are required for the formation of highly durable spores (Fig. 1).

In *M. xanthus* fruiting bodies, about 20% of the cells form spores. Other cells become non-reproductive peripheral rods (a persister-like state, approximately 30%) or undergo lysis (approximately 50%) [16]. The extent of cell lysis depends on environmental conditions, with the availability of nutrients likely to regulate the extent of cell death [73]. Lysed myxococcal cells release building materials such as protein S, which self-assembles on the spore surface, contributing to spore durability. The absence of protein S affects the spore coat thickness, as seen in glycerol-developed spores that lack protein S on their surface [72, 74]. The death of part of the cell population within the fruiting body thus provides nutrients and structural components that allow the remaining cells to survive and differentiate into robust spores.

Fruiting body development and sporulation in *M. xanthus* are controlled by specific extracellular signals like the A signal (a mixture of amino acids released from cells) and the C signal (a membrane-bound peptide oriented to the extracellular space), along with associated regulatory pathways [75–77]. Cell death is part of this developmental program and is referred to as PCD, considered an example of altruistic cell lysis [16, 78, 79]. The exact mechanism of PCD in *M. xanthus* remains unclear, but two strategies are considered: the regulated production of autocides (lipidic compounds like fatty acids and phosphatidylethanolamines) leading to cell membrane destabilization and eventual cell lysis [80, 81], and the involvement of the typical toxin/antitoxin system MazF and MrpC [73, 82].

Another example of bacterial cell death is the formation of aerial hyphae and sporulation in *Streptomyces* (Fig. 1), triggered by starvation-induced signals such as A-factor (an extracellular γ-butyrolactone) in *S. griseus*. This signal leads to the coordinated initiation of aerial hyphae formation in vegetative mycelium, followed by spore formation. The process involves a complex signaling cascade of intracellular regulators (including sigma factors that control RNA polymerase specificity) and extracellular factors that alter the surface hydrophobicity of aerial hyphae and spores (e.g., SapB hydrophobic protein) [24, 83]. During the initiation of aerial hyphae formation, part of the vegetative mycelium dies, and it is expected that the released nutrients feed the aerial mycelium. Supporting this, radiolabeling experiments have shown that part of the substrate mycelium biomass is cannibalized by the aerial mycelium [84, 85]. A proteomics analysis of dying *S. coelicolor* cells protoplasts revealed the expression of enzymes involved in membrane and cell content degradation (such as various proteases and hydrolases) [86]. Interestingly, further studies investigating the transition between vegetative and aerial mycelium revealed an alternating patterned dead and live cell regions within the mycelium, as well as spatial diversification between live and dead cells [87, 88]. These findings suggest that *Streptomyces* employ cell death as an integral mechanism during the development of its spatially organized multicellular structures, contributing to differentiation and resource allocation for aerial hyphae and spore formation.

## Extracellular matrix composition in biofilms: the influence of cell death

### ECM formation, composition, and role in microbial biofilms

A typical feature of all yeast and bacterial biofilms, both experimental and natural, is the presence of an extracellular matrix (ECM), an extracellular, cell-free mass that covers cells in the biofilm and fills intercellular spaces. The ECM has various functions, including protecting biofilm cells from desiccation and external toxic substances and antibiotics, serving as a storage reservoir for nutrients, and contributing to biofilm structure formation [27, 30, 89–91]. ESEM has visualized ECM in *S. cerevisiae* colony biofilms, showing intercellular cavities and channels that could be important for nutrient and waste flow, similar to channels in *B. subtilis* colony biofilms [30, 92]. The ECM composition [89, 93] varies depending on the type of biofilm and the organism, and it is also influenced by the environment in which the biofilm develops. The ECM typically contains proteins, carbohydrates (mainly polysaccharides), and may also contain nucleic acids, mainly extracellular DNA (eDNA), which form the backbone of some biofilm types and could contribute to biofilm resilience [89, 94, 95].

**Fig. 3:**
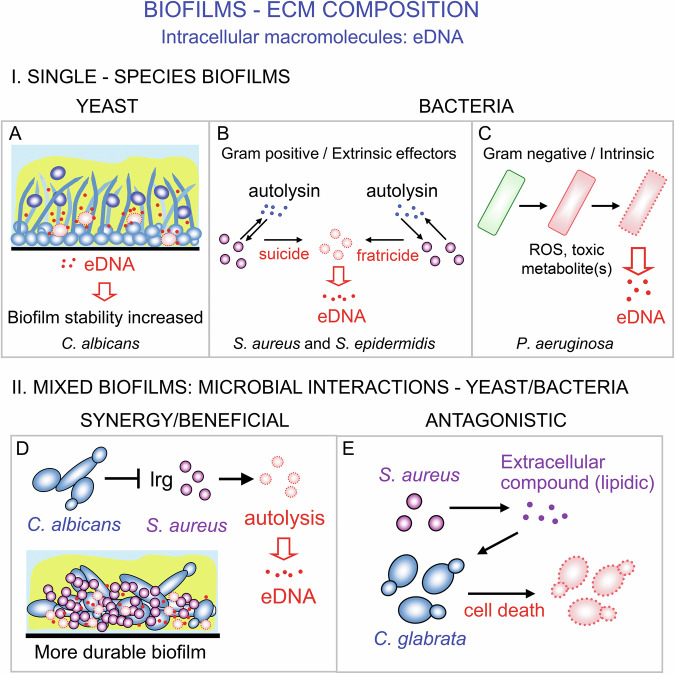
Contribution of cell death to ECM composition in different types of biofilms. **A**–**C** Examples of cell death in biofilms of yeast and of bacterial species leading to the release of extracellular DNA (eDNA), which becomes an integral component of the extracellular matrix (ECM, yellow) and enhances biofilm stability and resistance. **A** Dying cells (dashed red circles) release eDNA (dots), and vital cells (blue structures) in *C. albicans* biofilms. **B** eDNA (dots) released by G+ bacteria during the dying process (violet circles → dashed red circles), whereby cell death is triggered by external factors such as autolysins (blue dots). This occurs either through a suicide mechanism (autolysis induced by a cell’s own autolysin) or a fratricide mechanism (a cell is killed by the autolysin produced by a genetically related kin cell). **C** eDNA (dots) released from G- bacteria during the dying process (green → dashed red), whereby cell death is induced by internal factors such as toxic metabolites that elevate ROS levels and cause cell death. **D**, **E** Two distinct types of interactions in mixed biofilms of *S. aureus* (violet) with two different yeast pathogens (*C. albicans* and *C. glabrata*) (blue). **D** *C. albicans*-induced *S. aureus* cell lysis (dashed circles), which occurs through inhibition of the bacterial negative regulator of autolysis (lrg). **E** *S. aureus*-induced cell death of *C. glabrata* (dashed red), leading to its elimination from the biofilm. Purple dots, an unidentified extracellular compound triggering *C. glabrata* cell death.

The ECM can be formed through multiple mechanisms, including the secretion of proteins and carbohydrates, synthesis by cell wall and extracellular enzymes, and the release of some components from dead cells (Fig. 3). The role of cell death as a source of ECM components is mostly discussed concerning eDNA and proteins that normally function intracellularly and do not contain secretory sequences [96]. An increasing number of these proteins (both bacterial and yeast) are classified as moonlighting proteins, capable of performing independent functions in different cellular compartments or extracellular spaces [97, 98]. Compelling evidence indicates that these proteins also function in adhesion and biofilm stability [98–100]. A logical assumption is that these moonlighting proteins, along with eDNA, become part of the ECM by being released from lysed cells. However, lysis-independent mechanisms of eDNA and moonlighting protein release by atypical secretion or vesicle transport have also been demonstrated [98].

### ECM and cell death in yeast biofilms

The ECM is a key component in biofilms of different yeast species, including *S. cerevisiae* and *Candida* spp., providing a range of protective and structural functions [30, 90, 91]. The presence of eDNA and eRNA has been demonstrated in biofilms of clinical isolates of the yeast *Rhodotorula mucilaginosa* [101], and eDNA has been found in biofilms of *C. albicans*, where it contributes to the stabilization of the biofilm structure. It is assumed that dead cells are the source of this eDNA (Fig. 3A) [102, 103]. Additionally, intracellular proteins, including those classified as moonlighting proteins, have been identified in the ECM of *C. albicans* biofilms [93]. Unfortunately, no systematic research has been conducted to identify yeast cell death within biofilms. This is partly due to the fact that only a few studies have investigated the internal architecture of yeast biofilms (both colony and solid-liquid interphase types), and few existing approaches allow investigation of native biofilms at the cellular level.

### ECM and cell death in bacterial biofilms

Numerous examples illustrate the roles of cell death in biofilm ECM-formation by bacteria. Bacterial autolysis (cell suicide) and allolysis (cell death induced by factors released from differentiated cells of the same species) are thought to be the main mechanisms for releasing eDNA in biofilms of Gram-positive and Gram-negative bacteria (Fig. 3B, C) [96].

Gram-positive bacteria, including clinically relevant staphylococcal species *S. aureus* and *S. epidermidis*, frequently produce peptidoglycan-cleaving enzymes known as autolysins (e.g., *S. aureus* Atl). Autolysins play crucial roles in biofilm formation by damaging their cell wall peptidoglycan integrity or triggering the death of neighboring sister cells (a process known as fratricide) (Fig. 3B) [104–107]. Although numerous autolysins and other bacteriocins have been identified, the molecular mechanisms have been elucidated for only a few. For example, in the biofilm of *Enterococcus faecalis*, eDNA release occurs through a fratricidal mechanism [108]. The death of lysis-prone sibling cells is triggered by predatory cells of the same population via the gelatinase GelE and the autolysin AtlA. The predatory cells are protected by an immune mechanism involving the protease SprE, which modifies AtlA.

In Gram-negative *Pseudomonas aeruginosa*, autolysis can be triggered by autointoxication from the increased production of toxic metabolites (e.g., phenazine and pyocyanin), a burst of reactive oxygen species (ROS) production induced by 2-n-heptyl-4-hydroxyquinoline N-oxide (HQNO), or by prophage activation (Fig. 3C) [96]. Bacterial cell autolysis is often controlled by quorum-sensing, a form of cell-cell communication mediated by small secreted molecules such as hydroxyl-alkyl-quinolones and homoserine-lactone, which enable cells to coordinate their behavior [109]. For example, HQNO, a hydroxyquinolone and homoserine-lactone quorum-sensing-regulated molecule, triggers bacterial cell death by inhibiting the Qi site of the cytochrome bc1 complex of the respiratory chain, leading to ROS production, loss of membrane integrity, and autolysis [110]. Interestingly, HQNO also inhibits the cytochrome bc1 complex in mitochondria of eukaryotic cells [111], which leads to opening mitochondrial permeability transition pores, triggering the release of mitochondrial contents and initiating cell death [112, 113]. In both cases, the process begins with the inhibition of the Qi site of the cytochrome bc1 complex and can be inhibited by cyclosporin A (an inhibitor of eukaryotic mPTP pores), resulting in the release of intracellular content to the medium (bacteria) or mitochondrial content to the cytosol (eukaryotic cells) [110].

### Cell death in mixed-species biofilms

In natural environments and mammalian host tissues, biofilms are typically composed of diverse microbial species (e.g., bacteria and yeast) that engage in various interactions. These interactions can be synergistic or antagonistic, and cell death can occur in both scenarios (Fig. 3).

One example is the pathogenic bacterium *S. aureus*, which can form biofilms with two different yeast pathogens, *C. albicans,* and *C. glabrata*, each resulting in different outcomes of bacteria-yeast interactions (Fig. 3D, E) [114, 115]. The interaction between *S. aureus* and *C. albicans* is synergistic, leading to the formation of more robust and resistant mixed biofilms that are beneficial for both bacterial and yeast cells (Fig. 3D) [115]. This interaction induces the lysis of some bacterial cells, leading to the release of eDNA, which is then incorporated into the ECM. eDNA presence increases the resilience of mixed biofilms compared to single-species biofilms of *S. aureus* and *C. albicans*. In mixed biofilms, bacterial lysis is expected to result from the inhibition of the bacterial lrgAB operon by an unidentified signal from *C. albicans* [115]. This is supported by decreased lrgA and lrgB expression in mixed biofilms and the increased accumulation of eDNA in the ECM of lrgB mutants (similar to the presence of *C. albicans*). LrgA and LrgB have been predicted to function as antiholins (acting against CidA holin), based on various experiments showing their repressive role in cell lysis, including increased murein hydrolase activity in lrgAB mutants [116, 117]. However, a recent study provided evidence that LrgA and LrgB may function as holins, though less prominently than CidA [118]. More studies are needed to clarify the conflicting evidence regarding LrgAB functions. A key point in mixed biofilms would be identifying the *C. albicans* signal that induces lysis and understanding its mechanism of action on lrgAB.

*S. aureus* also exhibits an antagonistic interaction in mixed biofilms with the yeast *C. glabrata*, leading to the death and elimination of the yeast from the biofilm (Fig. 3E) [114]. Under experimental mixed biofilm conditions, the presence of *S. aureus* reduces the viability of *C. glabrata*, inducing ROS production, chromatin condensation, and DNA breaks in the yeast. The exact mechanism remains unclear, but a heat-resistant extracellular factor ( <3 kDa) produced by *S. aureus* likely plays a role in killing *C. glabrata*. The cell-free extract of. *S. aureus* inhibits the formation of biofilms by *C. glabrata*, *C. parapsilosis*, and *C. krusei*, but not by *C. albicans*, *C. neoformans*, and *S. cerevisiae* [114].

## The role of cell death in morphogenesis of spatial microbial structures

**Fig. 4:**
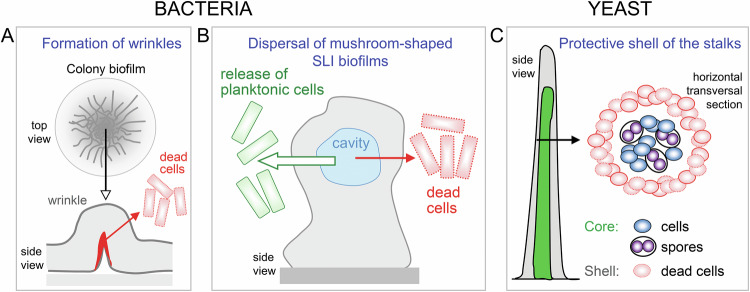
Contribution of cell death to the development of spatial microbial structures. **A** In *B. subtilis* colony biofilms, cell death plays a role in wrinkle formation. Top view of the entire colony biofilm with wrinkles schematically depicted as gray lines; the side view shows a magnified section of a single wrinkle with the localization of dead cells marked in red. **B** In mushroom-shaped biofilms of Gram-negative bacteria, cell death (red solid arrow) contributes to the formation of internal cavities (blue) and facilitates the release of planktonic cells (open arrow). **C** In yeast stalk cross-section, dying cells (outer ring) localize to the outer layers, forming a protective envelope around the stalk, while vital cells (blue) and sporulating (purple) cells localize to the inner core (green in the left image). Left, side view of the stalk with the position of the inner core; right, a horizontal section at the position indicated by the black arrow.

Cell death is an essential mechanism in metazoan morphogenesis, where a large portion of cells die during ontogeny. In addition to the scenarios discussed thus far, an intriguing question is whether cell death mechanisms occur during the development of spatially structured microbial communities that display visible structural complexity, such as various types of wrinkled biofilms (colony and SLI), or during formation of structures like stalks or fingers (Fig. 1). Although this question remains largely unanswered, there is evidence suggesting a possible role of cell death in the formation of wrinkles in bacterial colony biofilms and in dispersal of aged, mushroom-shaped bacterial biofilms, from which planktonic cells are released (Fig. 4) [119–121].

### Wrinkle formation in colony biofilms: a potential role for cell death

Colony biofilms formed by various wild strains of yeast (most studied in *S. cerevisiae*) and bacteria (most studied in *B. subtilis*) share structural similarities. These include surface wrinkles visible to the naked eye, microscopic channels that are thought to facilitate liquid flow, and an ECM that covers and connects the cells [30, 92]. Evidence suggests a role for cell death in the formation of wrinkles in *B. subtilis* colony biofilms. Dying cells were found at the base of *B. subtilis* 3-day-old biofilm wrinkles (Fig. 4A), leading to the hypothesis that localized cell death in areas of high cell density spatially concentrates lateral forces, promoting vertical biofilm bulging and wrinkle formation [119]. By locally altering cell density, the researchers were able to induce cell death and create artificial wrinkle patterns in biofilms [119]. The mechanism of this cell death is unknown.

In contrast to *B. subtilis*, no dead cells were observed at the base of wrinkles in *S. cerevisiae* colony biofilms during the observed timeframe of 24–72 h [27]. This raises questions about whether cell death may play a role in wrinkle formation in *S. cerevisiae* that has not yet been detected, or whether wrinkle formation occurs via entirely different mechanisms in *B. subtilis* (bacteria) and *S. cerevisiae* (yeast). Despite visible similarities in the morphology of the surface wrinkles, a key structural difference lies in how the wrinkles attach to the agar. *B. subtilis* colony biofilms are non-invasive, and grow entirely on the agar surface [122]. In contrast, *S. cerevisiae* colony biofilms develop invasive pseudohyphae at their base, which form massive “root-like” invasive structures within the agar [27]. As a result, *S. cerevisiae* biofilms consist of two distinct structural components: invasive “roots” embedded within the agar (absent in *B. subtilis* biofilms) and aerial wrinkles above the agar surface (Fig. 1). The proposed model for wrinkle formation in *S. cerevisiae* biofilms involves two primary factors: (i) the anchoring of the structure to the agar at multiple sites by invasive cells, and (ii) mechanical bulging of the aerial part (composed of cells connected by adhesins), driven by cell growth and the expansion of the ECM through hydration [27, 33, 123]. *S. cerevisiae* wrinkle formation could therefore differ fundamentally from that of *B. subtilis*, at least in the early stages of biofilm development studied so far (up to 3–4 days). Further research is needed to determine whether cell death plays a role in later stages of *S. cerevisiae* biofilm growth and whether it has any physiological function during biofilm morphogenesis.

### Life cycle of SLI biofilms: impact of cell death on biofilm dispersal

Yeast and bacterial SLI biofilms develop on solid surfaces in liquid media, such as experimental biofilms adhered to microtiter plastic plates or at the surfaces of continuous culture flow channels. SLI biofilm development typically progresses through four main phases: cell adhesion, initiation of biofilm spatial growth and ECM production, biofilm maturation, and partial biofilm dispersion.

#### Cell death in bacterial SLI biofilms

Mature bacterial SLI biofilms often exhibit mushroom-shaped structures with internal cavities from which planktonic cells can be released during biofilm dispersal [124]. In these biofilms, cell autolysis is thought to allow neighboring viable bacterial cells within the cavity to detach first from the structure and then from the biofilm as planktonic cells (Fig. 4B). The mechanisms inducing cell lysis vary among bacteria and include the role of prophages (e.g., *Pseudomonas aeruginosa*, [121]) or the autotoxic protein AlpP (e.g., *Pseudoalteromonas tunicata*, [120]). Exopolysaccharide Psl-specific staining has shown the appearance of Psl-free matrix cavities with dead cells and eDNA in the center of *P. aeruginosa* biofilms. Cell lysis was accompanied by the release of ECM exopolysaccharide Psl. Deletion of genes that control autolysis (CidA/LrgAB, putative holin/antiholin) affected the formation of cavities and biofilm dispersal in opposite ways, according to their predicted antiholin/holin functions—increasing in lrgAB mutants and decreasing in cidA mutants [125]. An example of quorum-sensing involvement in biofilm dispersal is the quorum-sensing factor AI-2-mediated prophage induction and upregulation of genes involved in cell lysis (endolysin, holin), resulting in the dispersal of *Enterococcus faecalis* biofilms [126].

In addition to cell death, other mechanisms can contribute to the release of planktonic cells from mature biofilms, including the degradation of the ECM by polysaccharide-degrading enzymes or proteases, the disruption of cell surface structures or the reduction of surface tension by surfactant molecules [124].

#### Cell death in yeast SLI biofilms

In contrast to bacteria, relatively few studies have examined the internal structure and dispersal mechanisms of yeast SLI biofilms, with *C. albicans* being the most studied yeast pathogen. To date, no dead cells have been directly observed in *C. albicans* or *S. cerevisiae* SLI biofilms, and the cell death can only be inferred based on eDNA and intracellular proteins present in the ECM of *C. albicans* biofilms [93, 103]. However, a comparative analysis of bacterial biofilms, where cell death facilitates the release of planktonic cells from cavities, and yeast SLI biofilms of different morphologies (*C. albicans* and *S. cerevisiae*) may provide insights where to search for dying cells in yeast biofilms. For example, the wild *S. cerevisiae* strain BR-F forms wrinkled biofilms with cell-free cavities similar to bacterial mushroom-shaped biofilms [28]. While the mechanism of planktonic cell release remains unclear, *S. cerevisiae* biofilm dispersal is induced by Cyc8, a repressor of biofilm formation, together with environmental glucose [26, 28].

In SLI biofilms of *C. albicans*, whose morphology differs from mushroom-shaped biofilms, the dispersal mechanisms are different. During steps 2 and 3 of the biofilm life cycle, spatially growing hyphae and/or pseudohyphae form, and dispersed planktonic cells (yeast-shaped cells) are expected to originate from the topmost hyphal layers. This dispersal involves a hyphae-to-yeast transition regulated by the *PES1* gene (pescadillo homolog) [127]. Environmental factors, such as the carbon source and pH of the media, also influence *C. albicans* biofilm dispersal [128].

Further research is needed to determine whether yeast biofilms, particularly those formed by *S. cerevisiae*, utilize cell death as a dispersal mechanism similar to bacteria. Understanding these processes could reveal new insights into the physiological functions of cell death in yeast biofilm dynamics.

### Cell death in yeast stalks

For completeness, we must mention the stalk structures formed by *S. cerevisiae*, one of the first structures in which specifically localized dead cells with a possible function in structure formation were identified (Fig. 4C). Yeast stalks are relatively thin vertical cylindrical structures that grow from small holes in dense agar [129] and are distinct from both the colonies and biofilms described above. These cylindrical structures, which are 0.5 to 3 cm high and 1 to 3 mm in diameter, consist of two main layers: an inner layer of living cells and spores, and a surface layer of vacuolated, dying cells with a thick cell wall. The authors hypothesize that the dead cells on the surface form a kind of shell that protects the inner cells [130], but no further studies have been conducted in this direction.

## Conclusions and perspectives

Current knowledge demonstrates the significance of cell death in spatially structured microbial communities. Dying cells can supply nutrients to long-lived subpopulations, contribute components for structure formation and protection, and play a role in the overall organization of the structure. However, it is noteworthy that much more studies and information are available on spatial structures formed by prokaryotic bacteria than on structures formed by eukaryotic yeast. At the same time, yeasts share greater similarities with higher eukaryotes in terms of cellular mechanisms and regulation, and their spatial structures arise not by aggregation as in the case of many bacterial structures, but through cell growth and differentiation similar to multicellular organisms.

One reason for the relative lack of information on cell death in yeast communities is the absence of adequate tools to identify dying cells directly in situ within spatial structures. In most identified cases, only a small, specifically localized fraction of cells undergo death, making whole-structure analyses insufficient and potentially compromised by methodological artifacts, such as the disruption of weakened dying cells during their separation from the structure. It is therefore crucial to develop techniques that, together with microscopy, allow the identification and observation of individual stages of cell death within the structure. Understanding the position and timing of cell death could then lead to the development of micromanipulation techniques for the rapid isolation of dying cells for further analysis, such as through OMICS tools.

Beyond the fundamental understanding of microbial community formation mechanisms, there are also practical reasons for identifying cell death mechanisms. In particular, understanding cell death in biofilms of yeast pathogens could hold promise for the development of therapeutic strategies aimed at inducing cell death and thereby eliminating parts of, or even entire, biofilms.
